# Supplementary material for: Absence of HTATIP2 Expression in A549 Lung Adenocarcinoma Cells Promotes Tumor Plasticity in Response to Hypoxic Stress
Source: Cancers (Basel). 2020 Jun 11;12(6):1538. doi: 10.3390/cancers12061538 (PMC7352940; doi:10.3390/cancers12061538)
Supplement: Supplementary file 1 [file cancers-12-01538-s001.pdf]

cancers

MDPI

Supplementary Materials

Absence of HTATIP2 Expression in A549 Lung Adenocarcinoma Cells Promotes Tumor Plasticity in Response to Hypoxic Stress

Minghua Li, Jing Li, Xiaofang Guo, Hua Pan and Qingyu Zhou

Table S1. Primary antibodies used in Western blot analyses.

Protein	Molecular Size (kDa)	Manufacturer	Catalog Number
Akt	60	Cell Signaling Technology	4691
Phospho-Akt (S473)	60	Cell Signaling Technology	4051
ERK1/2	42, 44	Cell Signaling Technology	4695
Phospho-ERK1/2 (T202/Y204)	42, 44	Cell Signaling Technology	4370
GSK3α/3β	51 (GSK3α), 46 (GSK3β)	Cell Signaling Technology	5676
Phospho-GSK3α/3β (S21/S9)	51, 46	Cell Signaling Technology	9331
MEK1/2	42	Cell Signaling Technology	8727
Phospho-MEK1/2 (S217/S221)	42	Cell Signaling Technology	9154
mTOR	289	Cell Signaling Technology	4517
Phospho-mTOR (S2448)	289	Cell Signaling Technology	2971
RSK1/2/3	90	Cell Signaling Technology	9355
Phospho-p90RSK (S380)	90	Cell Signaling Technology	11989
HIF1α	120	Cell Signaling Technology	36169
HIF2α	120	Cell Signaling Technology	59973
HTATIP2	28, 32	Cell Signaling Technology	14614
c-MYC	57-65	Cell Signaling Technology	18583
MCL-1	40	Cell Signaling Technology	5453
MXI-1	33	Thermo Fisher Scientific	PA5-71929
E-Cadherin	130	Cell Signaling Technology	3195
β-Catenin	92	Cell Signaling Technology	8480
Vimentin	50, 57	Cell Signaling Technology	5741
β-Actin	42	Cell Signaling Technology	3700

Note: Individual primary antibodies were used at a dilution of 1:1000 in Western blot analysis.

Table S2. Primers used in the quantitative real-time PCR.

Human Gene	Forward 5′ → 3′	Reverse 5′ → 3′
HTATIP2	CGGAGGGATTTGTTCGTGTT	TGAAATGTTTGCACCCTCCA
HIF1α	AAGGTATTGCACTGCACAGG	TCAGCACCAAGCAGGTCATA
HIF2α	TCTACCATGCGCTAGACTCC	CCGGTACTGGCCACTTACTA
c-Myc	ATTCTCTGCTCTCCTCGAC	CTTCCTCATCTTCTTGTTCCTC
MXI1	CGGCACACAACACTTGGTT	AAATTCTCGAGCTGGTGCTG
VEGFA	AATGACGAGGGCCTGGAG	GCCTTGGTGAGGTTTGATCC
PDGFB	CGAGTTGGACCTGAACATGA	CTCAGCAATGGTCAGGGAAC
GLUT-1	ATTGGCTCCGGTATCGTCAAC	GCTCAGATAGGACATCCAGGGTA
GAPDH	GGACTCATGACCACAGTCCA	AGGCAGGGATGATGTTCTGG

Table S3. Concentrations of individual metabolites in A549 and A549shHTATIP2 tumors determined by the LC-MS/MS.

	Component Name	Metabolite Concentration (pmol/mg tissue)			
		A549 (*N* = 4)		A549shHTATIP2 (*N* = 6)	
		Mean	SD	Mean	SD
1	M001_L-Tryptophan	87.90	11.68	88.48	18.15
2	M002_L-Kynurenine	0.342	0.105	0.244	0.054
3	M003_Serotonin	0.074	0.034	0.057	0.029
4	M004_5-Hydroxyindoleacetic acid	0.302	0.072	0.484	0.315
5	M005_Kynurenic acid	0.037	0.053	0.005	0.005
6	M008_Succinic acid	18.358	5.107	22.304	3.960
7	M009_Fumaric acid	85.601	8.452	85.283	11.963
8	M012_L-Malic acid	340.92	25.99	382.26	56.63
9	M013_Succinyl-CoA	0.811	0.310	0.408	0.228
10	M015_Acetyl-CoA	0.180	0.082	0.078	0.045
11	M016_2-ketoglutaric acid	3.695	1.190	3.563	2.718
12–14	M019_Fructose 6-phosphate+M106_Glucose 6-phosphate+M051_Glucose 1-phosphate	1312.4	460.1	1071.9	220.1
15	M020_Fructose 1,6-bisphosphate	621.46	88.70	791.40	221.92
16–17	M021_2-Phosphoglyceric acid+M305_3-Phosphoglyceric acid	31.792	7.572	41.442	19.019
18	M023_L-Valine_new	475.11	14.04	501.94	37.15
19	M024_Leucine(s)	645.718	53.426	619.828	62.997
20	M025_Histidine	399.568	55.322	369.052	133.617
21	M026_Phenylalanine	249.263	20.269	238.411	30.155
22	M027_Glutamine(s)	225.530	62.680	116.533	27.750
23	M028_Tyrosine	305.283	54.284	342.513	60.839
24	M029_Isoleucine(s)	272.931	25.549	285.365	17.459
25	M030_Threonine(s)	398.780	29.433	363.121	30.743
26	M031_L-Glutamic acid	1496.4	148.8	1638.8	220.1
27	M032_Arginine	87.835	11.626	83.393	13.570
28	M033_Lysine (s)	245.48	47.89	246.83	46.57
29	M034_S-Adenosylmethionine	2.821	0.607	1.771	0.632
30–31	M035_Dihydroxyacetone phosphate+M097	141.04	11.89	128.43	42.98
32	M036_Picolinic acid	193.05	22.67	173.34	21.81
33	M039_NADP	7.928	2.463	3.102	2.303
34	M041_NAD	1.286	0.520	0.789	0.367
35	M043_Dephospho-CoA	3.349	0.979	1.891	0.698
36	M046_3-Hydroxy-3-methylglutaryl-CoA	0.005	0.003	0.005	0.004
37	M047_Nicotinamide	188.11	19.95	170.27	20.83
38	M048_AICAR	0.781	0.852	0.671	0.274
39	M049_S-Adenosylhomocysteine	2.987	1.109	1.952	0.996
40	M050_Deoxyribose 5-phosphate	1.833	0.580	1.510	0.371
41	M052_dUMP	BLQ	BLQ	BLQ	BLQ
42	M054_Hypoxanthine	775.4	147.5	802.6	215.3
43	M055_D-Ribose 5-phosphate	53.932	26.756	32.204	14.282
44	M056_Glutathione	953.7	267.7	302.1	229.6
45	M057_Gluconic acid	111.38	16.15	100.72	22.25
46	M058_Uric acid (s)	83.85	20.38	106.85	51.00
47	M062_1-Methylhistidine	4.335	1.030	4.337	0.602
48	M063_1-Methylnicotinamide	11.114	1.881	12.931	1.989
49	M064_Gentisic acid	BLQ	BLQ	BLQ	BLQ
50	M065_Deoxycytidine	BLQ	BLQ	BLQ	BLQ
51	M068_2-Ketohexanoic acid_NEG	0.085	0.019	0.125	0.081
52	M069_5-Methoxytryptophan	BLQ	BLQ	BLQ	BLQ
53	M070_L-Acetylcarnitine	62.853	5.272	64.833	3.321
54	M071_N-Alpha-acetyllysine	0.640	0.421	0.369	0.189
55	M072 cis aconitic acid same as M007	0.761	0.231	0.841	0.359
56	M073_Adenosine	13.171	5.226	9.953	2.426
57	M074_Adenosine diphosphate ribose	1.483	1.707	0.330	0.092
58	M075_ADP	19.295	19.004	7.593	2.276
59	M076_Agmatine	18.330	5.102	9.474	3.452
60	M077_Aminoadipic acid	12.495	3.855	8.369	2.682

61	M078_aminolevulinic acid	BLQ	BLQ	BLQ	BLQ
62	M079_AMP	0.293	0.315	BLQ	BLQ
63	M080_Biotin	0.005	0.001	0.007	0.003
64	M082_Butyrylcholine	BLQ	BLQ	BLQ	BLQ
65	M083_Carnitine	36.445	7.146	43.063	7.138
66	M084_Chlorzoxazone	BLQ	BLQ	BLQ	BLQ
67	M086_Citrulline	73.034	26.724	72.747	14.748
68	M087_CMP	12.872	1.042	12.109	2.726
69	M089_Cytosine	BLQ	BLQ	BLQ	BLQ
70	M090_dAMP	1.005	0.275	0.637	0.411
71	M092_deoxyinosine	0.335	0.047	0.354	0.099
72	M093_Deoxyuridine	BLQ	BLQ	BLQ	BLQ
73	M096_DL-2-Aminooctanoic acid	0.205	0.061	0.238	0.092
74	M097_D-Glyceraldehyde 3-phosphate(s)	BLQ	BLQ	BLQ	BLQ
75	M099_FAD	10.691	1.655	9.005	1.639
76	M100_Flavone	BLQ	BLQ	BLQ	BLQ
77	M101_Folic acid	BLQ	BLQ	BLQ	BLQ
78	M105_glucosamine	BLQ	BLQ	BLQ	BLQ
79	M107_Glucuronic acid	4.506	0.932	3.230	0.934
80	M109_GMP	BLQ	BLQ	BLQ	BLQ
81	M112_Guanidoacetic acid	5.806	0.442	4.622	1.267
82	M113_Guanine	2.505	0.630	3.694	1.342
83	M114_Guanosine	27.268	8.151	12.883	4.559
84	M117_Homoserine (s)	4.209	0.221	4.592	0.598
85	M118_Hydroxyisocaproic acid	2.329	0.484	3.248	0.600
86	M121_Imidazole	0.174	0.062	0.319	0.154
87	M122_Imidazoleacetic acid	6.298	0.994	6.506	2.113
88	M123_IMP	BLQ	BLQ	BLQ	BLQ
89	M124_Indole	2.717	0.987	3.234	1.796
90	M125_Indole-3-carboxylic acid	BLQ	BLQ	BLQ	BLQ
91	M126_Indoleacrylic acid	0.004	0.003	0.006	0.004
92	M127_Lipoic acid	BLQ	BLQ	BLQ	BLQ
93	M128_L-Pipecolic acid (s)	20.168	6.540	28.865	7.279
94	M129_Methionine sulfoxide	3.376	1.152	3.087	0.823
95	M131_N-Acetyl-L-aspartic acid	17.231	2.170	21.174	5.888
96	M132_N-Acetyl-D-glucosamine	7.491	1.809	9.734	2.589
97	M133_N-Acetyl-L-alanine (s)	399.09	31.00	409.69	33.54
98	M134_N-Acetylornithine	2.779	0.137	1.192	0.546
99	M137_Nicotinic acid	BLQ	BLQ	BLQ	BLQ
100	M138_N-Methyl-D-aspartic acid	870.40	99.28	956.16	129.27
101	M140_Ornithine	19.427	15.835	27.617	11.935
102	M143_Pantothenic acid#	7.265	1.187	7.746	0.889
103	M144_Perfluoroheptanoic acid	BLQ	BLQ	BLQ	BLQ
104	M145_Phenyllactic acid	269.91	34.49	288.49	45.09
105	M146_Phenylpropiolic acid	BLQ	BLQ	BLQ	BLQ
106	M149_4-Hydroxybenzoic acid	0.926	0.766	0.596	0.445
107	M150_L-Proline	430.49	63.53	654.77	98.46
108	M151_Purine	0.058	0.010	0.058	0.026
109	M152_Pyridoxal 5'-phosphate	4.067	1.819	2.888	1.428
110	M153_Pyridoxamine	0.028	0.004	0.015	0.003
111	M154_pyridoxine	BLQ	BLQ	BLQ	BLQ
112	M155_Pyroglutamic acid +M128	12.084	3.003	14.742	4.588
113	M157_Flavin Mononucleotide	0.329	0.136	0.383	0.302
114	M158_Shikimic acid	BLQ	BLQ	BLQ	BLQ
115	M160_Sorbitol	95.696	26.300	139.777	53.447
116	M161_Taurine	3055.0	182.5	3265.9	498.6
117	M162_Thiamine	7.002	2.638	2.878	0.688
118	M163_Thymidine	0.825	0.108	0.436	0.112
119	M164_Thymine	2.014	0.223	3.104	0.299
120	M166_Uridine diphosphate-N-acetylglucosamine	177.68	13.87	115.14	56.92
121	M167_Uridine 5'-monophosphate	5.330	5.601	2.751	2.038
122	M168_Uracil	301.72	49.00	339.26	56.01
123	M169_Uridine	487.91	115.17	571.41	79.86

124	M170_Xanthine	312.38	43.88	303.09	42.25
125	M171_Xanthosine	6.076	1.676	7.058	2.288
126	M172_Xanthylic acid	#DIV/0!	#DIV/0!	#DIV/0!	#DIV/0!
127	M173_Xanthurenic acid	0.027	0.013	0.020	0.017
128	M174_Adenosine triphosphate	20.041	15.950	7.126	6.692
129	M175_Cytidine triphosphate	BLQ	BLQ	BLQ	BLQ
130	M176_Guanosine triphosphate	6.108	2.812	4.141	0.999
131	M177_Uridine triphosphate	1.915	0.776	1.274	0.689
132	M178_dATP	BLQ	BLQ	BLQ	BLQ
133	M179_dCTP	BLQ	BLQ	BLQ	BLQ
134	M180_dGTP	BLQ	BLQ	BLQ	BLQ
135	M181_dTTP	BLQ	BLQ	BLQ	BLQ
136	M182_L-Cysteine	245.29	65.47	723.48	509.06
137	M183_L-Alanine	145.43	16.90	173.60	28.29
138	M184_L-Asparagine	39.222	3.985	39.221	10.213
139	M185_Glycine	15.053	8.288	17.873	12.685
140	M186_L-Aspartic acid	279.37	20.37	250.67	46.82
141	M187_Argininosuccinic acid	4.306	0.657	4.461	0.903
142	M188_Sarcosine (s)	817.64	88.15	825.30	76.61
143	M189_Dimethylglycine+M191	34.291	2.827	35.750	6.657
144	M190_Betaine	36.303	7.987	51.894	10.322
145	M191_Choline	130.739	13.517	133.092	24.465
146	M192_Cystathionine	24.292	9.990	23.179	17.877
147	M194_Urea	3527.5	406.5	4229.4	588.4
148	M195_Cytidine	3.007	1.073	2.352	0.367
149	M196_CDP	BLQ	BLQ	BLQ	BLQ
150	M197_GDP	3.475	1.538	2.967	1.074
151	M198_UDP	BLQ	BLQ	BLQ	BLQ
152	M200_dCMP	0.334	0.166	0.486	0.056
153	M201_dCDP	BLQ	BLQ	BLQ	BLQ
154	M202_dGMP	BLQ	BLQ	BLQ	BLQ
155	M203_dGDP	19.05	20.52	7.44	2.37
156	M204_dTMP	BLQ	BLQ	BLQ	BLQ
157	M205_dTDP	BLQ	BLQ	BLQ	BLQ
158	M206_uridine diphosphate glucose	16.549	7.608	17.744	7.090
159	M207_ADP-glucose_new	0.593	0.193	0.825	0.380
160	M211_Maleic acid_NEG	BLQ	BLQ	BLQ	BLQ
161	M216_2-Aminobenzoic acid	0.018	0.026	0.010	0.006
162	M217_p-Aminobenzoic acid	BLQ	BLQ	BLQ	BLQ
163	M219_2-Oxo-4-methylthiobutanoic acid	BLQ	BLQ	BLQ	BLQ
164	M220_2-Pyrocatechuic acid	BLQ	BLQ	BLQ	BLQ
165	M221_L-Dihydroorotic acid	64.41	22.77	63.73	13.34
166	M224_4-Pyridoxic acid	0.013	0.004	0.013	0.002
167	M225_2-Keto-D-gluconic acid	0.293	0.090	0.220	0.068
168	M226_D-Erythrose 4-phosphate	116.24	40.33	98.90	17.68
169	M228_Inosine	BLQ	BLQ	BLQ	BLQ
170	M229_D-Sedoheptulose 7-phosphate	80.191	32.160	50.023	6.467
171	M230_N-Acetyl-glucosamine 1-phosphate	40.93	13.63	59.28	30.74
172	M231_Cyclic AMP	0.244	0.030	0.351	0.079
173	M232_Sucrose	0.298	0.175	0.564	0.498
174	M238_Thiamine pyrophosphate	BLQ	BLQ	BLQ	BLQ
175	M239_Adenosine phosphosulfate	BLQ	BLQ	BLQ	BLQ
176	M241_Cholesterol sulfate	7.079	3.517	8.418	4.468
177	M242_Citicoline	42.62	19.02	24.82	19.09
178	M243_Taurodeoxycholic acid	BLQ	BLQ	BLQ	BLQ
179	M245_Coenzyme A	4.024	1.789	0.992	0.799
180	M246_Propionyl-CoA	0.008	0.001	0.003	0.002
181	M247_3-Hydroxybutyryl-CoA	BLQ	BLQ	BLQ	BLQ
182	M248_L-2-Hydroxyglutaric acid	9.814	4.139	10.942	2.173
183	M249_Ethanolamine	129.16	37.90	77.36	24.76
183	M249_Ethanolamine_new	156.48	46.36	77.32	25.70
184	M251_Gamma-Aminobutyric acid	5.454	0.983	5.336	1.785
185	M254_Creatinine	9.993	10.026	5.315	0.689

186	M255_Creatine (s)	1143.39	1200.07	574.08	92.33
187	M256_N-Acetylputrescine	0.178	0.096	0.259	0.056
188	M257_4-Hydroxyproline(s)	8.543	2.065	7.117	1.688
189	M258_Adenine	1.298	0.659	0.712	0.209
190	M259_L-Homocysteine	23.18	14.10	116.01	49.86
191	M260_L-Histidinol	BLQ	BLQ	BLQ	BLQ
192	M262_Phosphorylcholine	861.95	113.96	743.24	140.33
193	M264_N-Acetylglutamine	0.235	0.031	0.259	0.039
194	M265_N-Acetylglutamic acid	1.952	0.260	2.357	0.566
195	M266_Asymmetric dimethylarginine	5.338	0.735	6.138	2.318
196	M267_L-Cystine#	0.072	0.048	6.003	8.114
197	M268_Deoxyadenosine (s)	0.093	0.044	0.020	0.011
198	M269_AICA-riboside	0.026	0.004	0.053	0.027
199	M271_7-Methylguanosine	BLQ	BLQ	BLQ	BLQ
200	M272_beta-nicotinamide D-ribonucleotide	0.020	0.006	0.009	0.005
201	M273_Riboflavin	0.396	0.093	0.655	0.376
202	M276_3,5-Diiodothyronine#	BLQ	BLQ	BLQ	BLQ
203	M277_Putrescine	19.309	6.516	14.988	3.664
204	M278_Spermidine	46.98	14.69	68.72	19.32
205	M279_Spermine	62.17	19.48	87.20	39.27
206	M281_Alpha-Hydroxyisobutyric acid	BLQ	BLQ	BLQ	BLQ
207	M282_4-Hydroxyphenyl acetic acid	BLQ	BLQ	BLQ	BLQ
208	M289_Dimethylamine	BLQ	BLQ	BLQ	BLQ
209	M290_Glycocholic acid	BLQ	BLQ	BLQ	BLQ
210–212	M291_Glycoursodeoxycholic acid +M297 Glycodeoxycolic acid+M313 Glycochendeoxychlate	BLQ	BLQ	BLQ	BLQ
213	M293_Hippurate	1.031	0.588	1.050	0.947
214–216	M294_Hyodeoxycholic acid same as M284_Chenodeoxycholic acid M303_NEG	BLQ	BLQ	BLQ	BLQ
217–218	M297 Glycodeoxycolic acid+M313 Glycochendeoxychlate	BLQ	BLQ	BLQ	BLQ
219	M299_Taurochenodesoxycholic acid	BLQ	BLQ	BLQ	BLQ
220	M300_Taurocholic acid	0.053	0.035	0.098	0.030
221	M302_Taurolithocholic acid	BLQ	BLQ	BLQ	BLQ
222	M303_Ursodeoxycholic acid	BLQ	BLQ	BLQ	BLQ
223	M306_Thiamine monophosphate	BLQ	BLQ	BLQ	BLQ
224	M307_2'-Deoxyguanosine	1.060	0.255	0.971	0.339
225	M308_Malonyl CoA	0.021	0.027	0.003	0.001
226	M309_R-2-Hydroxy-2-phenylpropionic acid	BLQ	BLQ	BLQ	BLQ
227	M310_D-Citramalic acid	BLQ	BLQ	BLQ	BLQ
228	M311_L-Homocysteic acid	BLQ	BLQ	BLQ	BLQ
229	M312_Serine	315.55	28.78	287.36	45.71
230	M314_CDP-ethanolamine	22.201	7.006	25.850	5.831
231	M315_p-cresyl sulfate	0.014	0.005	0.011	0.009
232	M316_Glycolithocholic acid	BLQ	BLQ	BLQ	BLQ
233	M317_Dihydro thymine	BLQ	BLQ	BLQ	BLQ
234	M318_Alpha-N-Phenylacetyl- L-glutamine	BLQ	BLQ	BLQ	BLQ
235	M319_N-Phenylacetylglycine	0.056	0.036	0.058	0.061
236	M327_Lithocholic acid_ss	BLQ	BLQ	BLQ	BLQ
237	M328_ Linoleyl Carnitine	1.813	0.457	2.844	1.545
238	M329_Valeryl-L carnitine	1.590	0.650	1.386	0.141
239	M330_Lauroyl-L carnitine	0.023	0.004	0.069	0.034
240	M331_Palmitoyl-L carnitine	1.200	0.575	2.145	0.852
241	M341_Myristoyl-L-carnitine	0.052	0.035	0.139	0.044
242	M342_Propionyl-L-carnitine	0.551	0.133	0.693	0.173
243	M343_Octanoyl-L-carnitine	0.016	0.008	0.035	0.011
244	M344_Decanoyl-L-carnitine	0.014	0.001	0.110	0.085
245	M345_Malonyl-L-carnitine	0.257	0.200	0.134	0.116
246	M346_Stearoyl-L-carnitine	1.406	0.774	3.067	1.375
247	M351_6-phosphogluconate	197.83	75.67	259.35	91.81
248	M352_GAR	BLQ	BLQ	BLQ	BLQ

Note: BLD means Below the limit of detection.

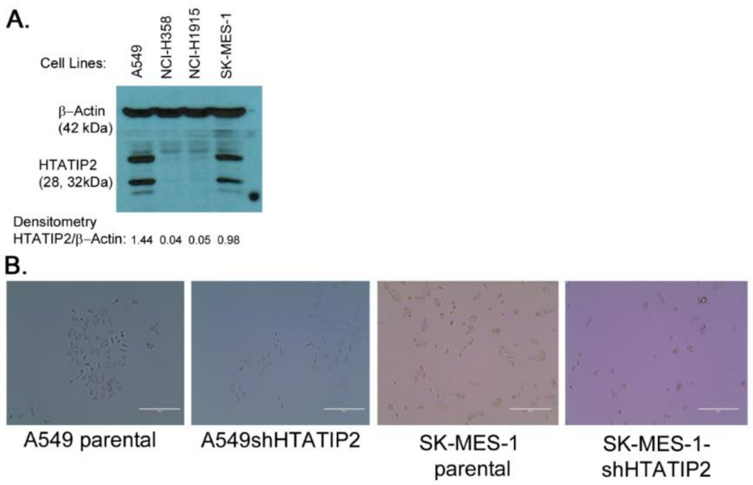

Figure S1. Expression of HTATIP2 Protein in A549, NCI-H358, NCI-H1915 and SK-MES-1 Cell Lines and Viability of HTATIP2 Knockdown Cell Lines. **(A).** Expression of HTATIP2 protein in A549, NCI-H358, NCI-H1915 and SK-MES-1 cell lines. Western blot images were quantified using the densitometric analysis. Relative immunoreactive band intensities are expressed as the ratio of the densitometric values for individual bands to the densitometric values for β-actin in the same lane. **(B).** Microscopic examination of the morphology of A549 and SK-MES-1 cells in culture after the cells were transduced with shRNA targeting HTATIP2 (shHTATIP2). Representative micrographs of A549 parental, A549shHTATIP2, SK-MES-1 parental and SK-MES-1-shHTATIP2 cell lines. Original magnifications, × 200. Scale bar, 200 μm.

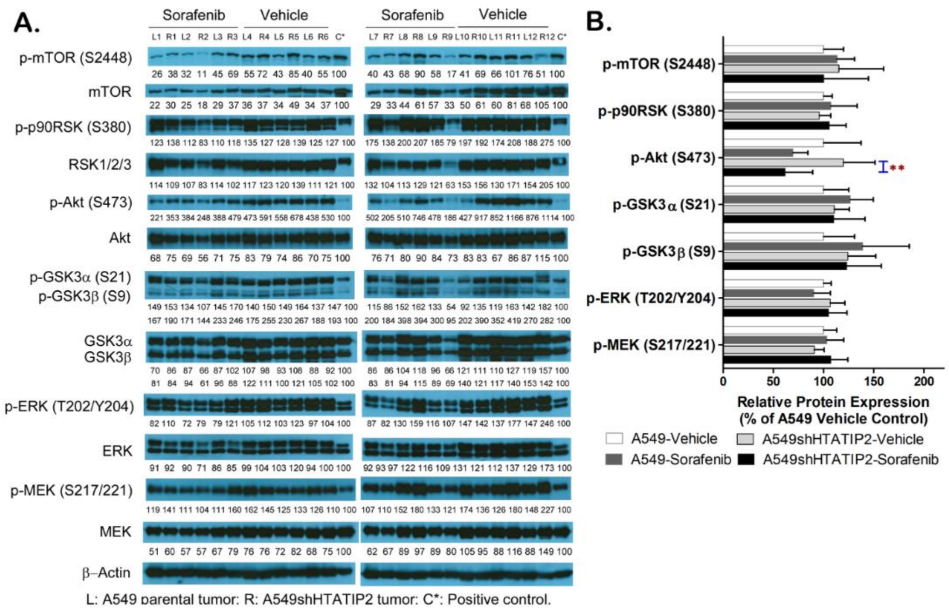

Figure S2. The absent expression of HTATIP2 had little impact on the main effectors of the MEK/ERK and Akt signaling pathways. **(A)**. Western blot images of the expression of total and phosphorylated mTOR, RSK1/2/3, Akt, GSK3α/3β, ERK1/2 and MEK1/2 in individual tumor samples. The positive control was used to compensate for both systematic and random errors from SDS-PAGE, membrane transfer, immunoblotting and chemiluminescence detection. **(B).** Quantification of Western blot images using the densitometric analysis. Expression levels of phosphorylated proteins are expressed as the ratio of phosphorylated-to-total species relative to the vehicle control A549 parental tumors. Data are presented as mean ± SD. Error bars are SD. ***p* < 0.01 compared between vehicle and sorafenib treatment in the same tumor type using the two-sample *t* test.

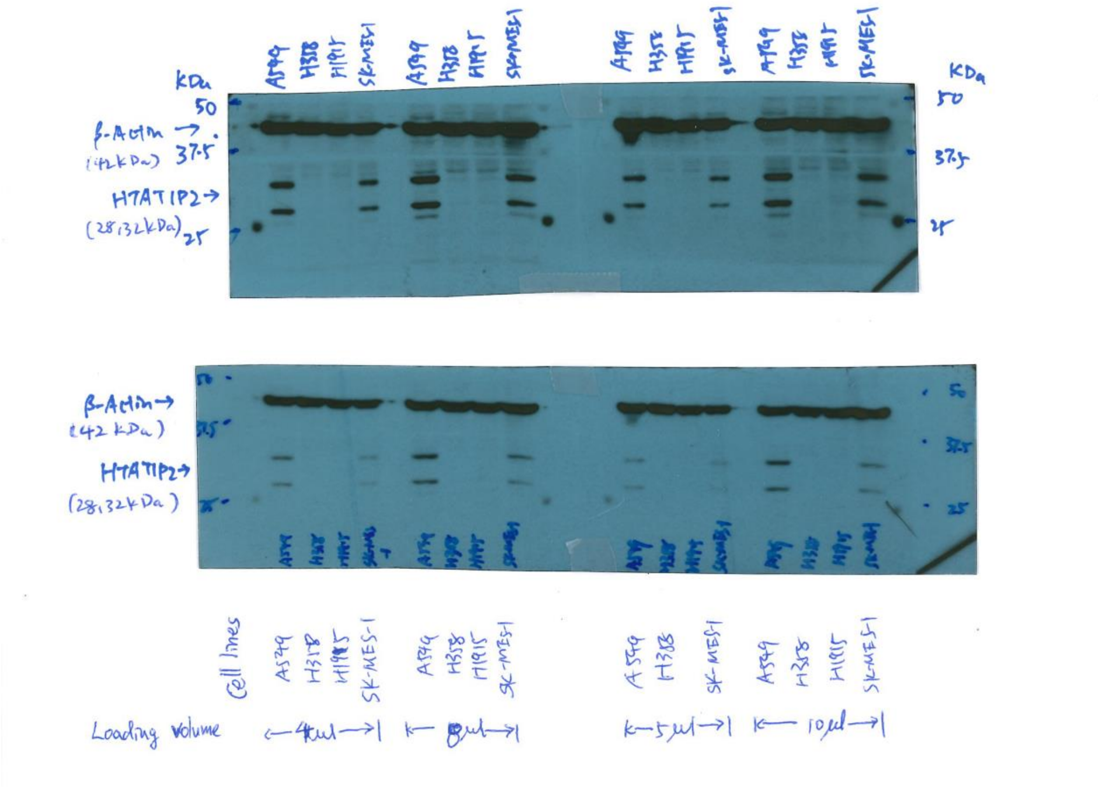

Supplemental Figure S3-1. Uncropped Western blot images showing HTATIP2 protein expression in different cell lines including A549, NCI-H358, NCI-H1915 and SK-MES-1 lung cancer cell lines.

Figure S3. Uncropped Western blot images.

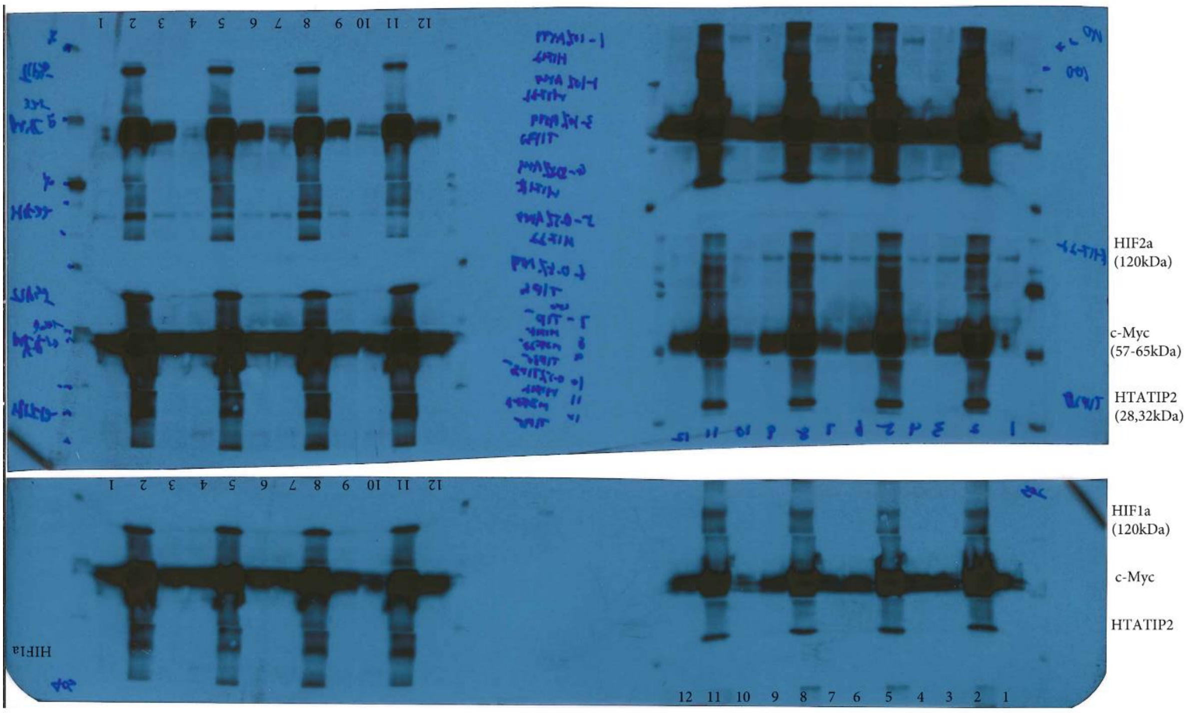

Supplemental Figure S3-2-1. Uncropped Wester blot images showing the results of the co-immunoprecipitation study. A549 cells were cultured under hypoxic condition (0.5% O2) for 24 hours in DMEM/F12 medium supplemented with either 10% or 0.5% FBS. Immunoprecipitation: 1, HIF1a; 2, HIF2a; 3, HTATIP2; 4, HIF1a; 5, HIF2a; 6, HTATIP2; 7, HIF1a; 8, HIF2a; 9, HTATIP2; 10, HIF1a; 11, HIF2a; 12, HTATIP2;

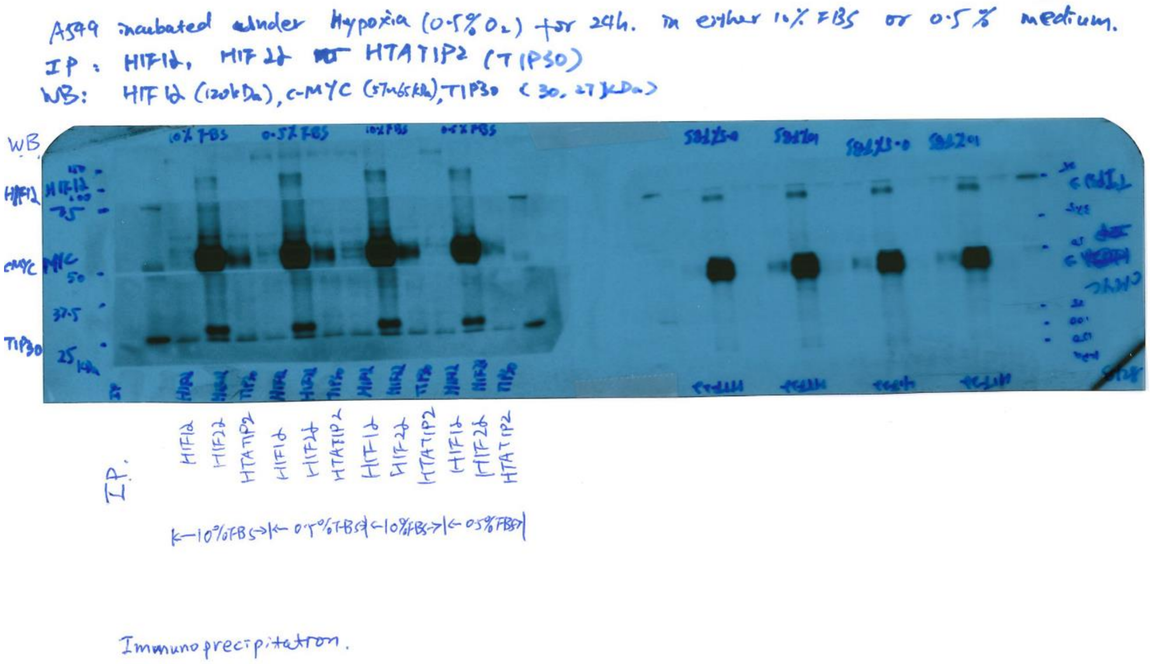

Supplemental Figure S3-2-2. Uncropped Wester blot images showing the results of the co-immunoprecipitation study. A549 cells were cultured under hypoxic condition (0.5% O2) for 24 hours in DMEM/F12 medium supplemented with either 10% or 0.5% FBS.

IN VITRO **HTATIP2 knockdown study**

A549shNT versus A549shHTATIP2 cell line under hypoxia (0.5% O2) and normoxia condition in the presence and absence of 10 μM sorafenib for 24 hours in DMEM containing 0.5% FBS with high glucose and L-glutamine and without sodium pyruvate. Samples were from three independent experiments [E1, E2 and E3]
From Left to Right:
(#1 ~4) A549shNT: Normoxia_control (NC), Normoxia_sorafenib (NS), Hypoxia_control (HC), Hypoxia_sorafenib (HS); (#5 ~8) A549shNT: Normoxia_control (NC), Normoxia_sorafenib (NS), Hypoxia_control (HC), Hypoxia_sorafenib (HS); #9: positive control.

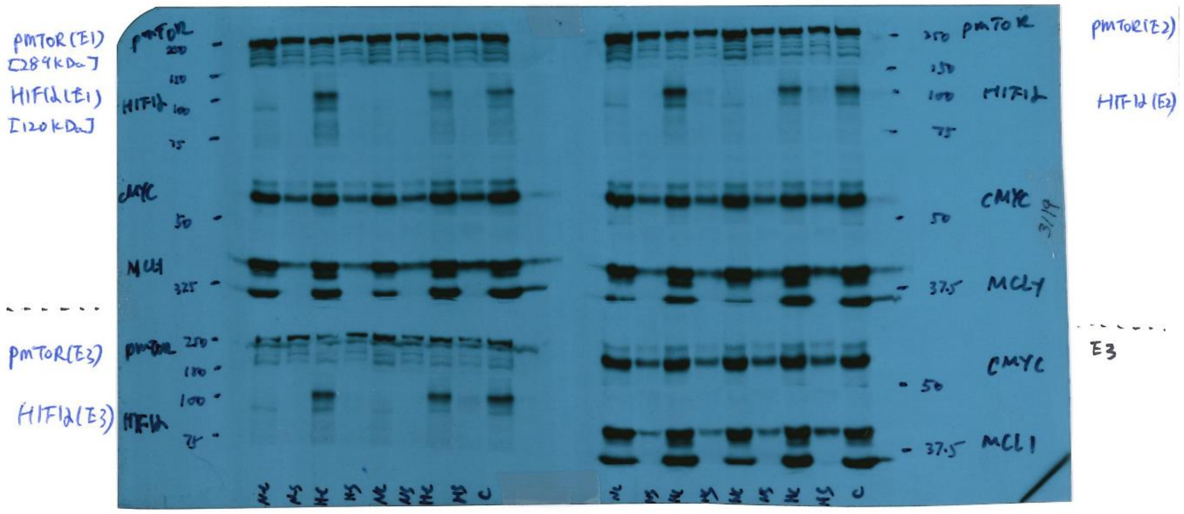

Supplemental Figure S3-3-1. Uncropped Western blot images showing the expression of phospho-mTOR (S2448; 289 kDa) and HIF1α (120 kDa) in A549shNT and A549shHTATIP2 cell lysates.

***IN VITRO* HTATIP2 knockdown study**

A549shNT versus A549shHTATIP2 cell line under hypoxia (0.5% O2) and normoxia condition in the presence and absence of 10 μM sorafenib for 24 hours in DMEM containing 0.5% FBS with high glucose and L-glutamine and without sodium pyruvate. Samples were from three independent experiments [E1, E2 and E3]
From Left to Right:
(#1 ~4) A549shNT: Normoxia_control (NC), Normoxia_sorafenib (NS), Hypoxia_control (HC), Hypoxia_sorafenib (HS); (#5 ~8) A549shNT: Normoxia_control (NC), Normoxia_sorafenib (NS), Hypoxia_control (HC), Hypoxia_sorafenib (HS); #9: positive control.

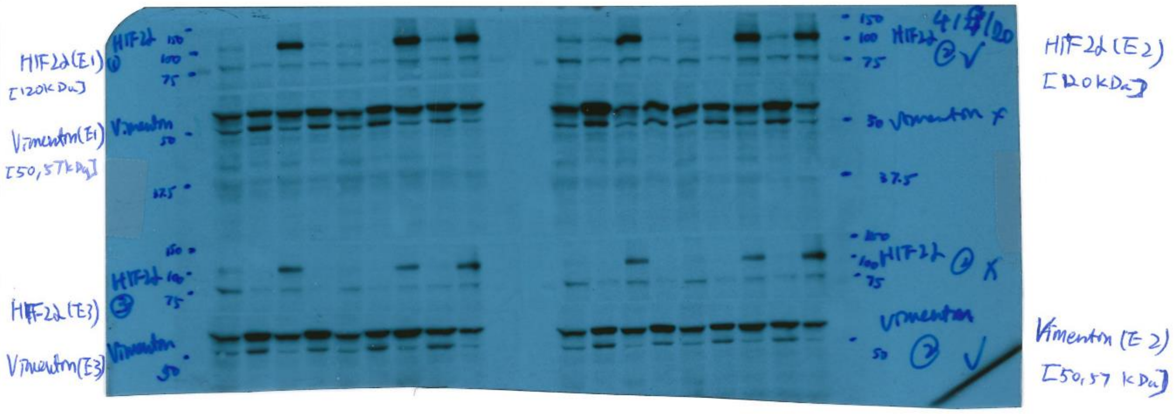

Supplemental Figure S3-3-2. Uncropped Western blot images showing the expression of HIF2α (120 kDa) and Vimentin (50, 57 kDa) in A549shNT and A549shHTATIP2 cell lysates.

IN VITRO **HTATIP2 knockdown study**

A549shNT versus A549shHTATIP2 cell line under hypoxia (0.5% O2) and normoxia condition in the presence and absence of 10 μM sorafenib for 24 hours in DMEM containing 0.5% FBS with high glucose and L-glutamine and without sodium pyruvate. Samples were from three independent experiments [E1, E2 and E3]
From Left to Right:
(#1 ~4) A549shNT: Normoxia_control (NC), Normoxia_sorafenib (NS), Hypoxia_control (HC), Hypoxia_sorafenib (HS); (#5 ~8) A549shNT: Normoxia_control (NC), Normoxia_sorafenib (NS), Hypoxia_control (HC), Hypoxia_sorafenib (HS); #9: positive control.

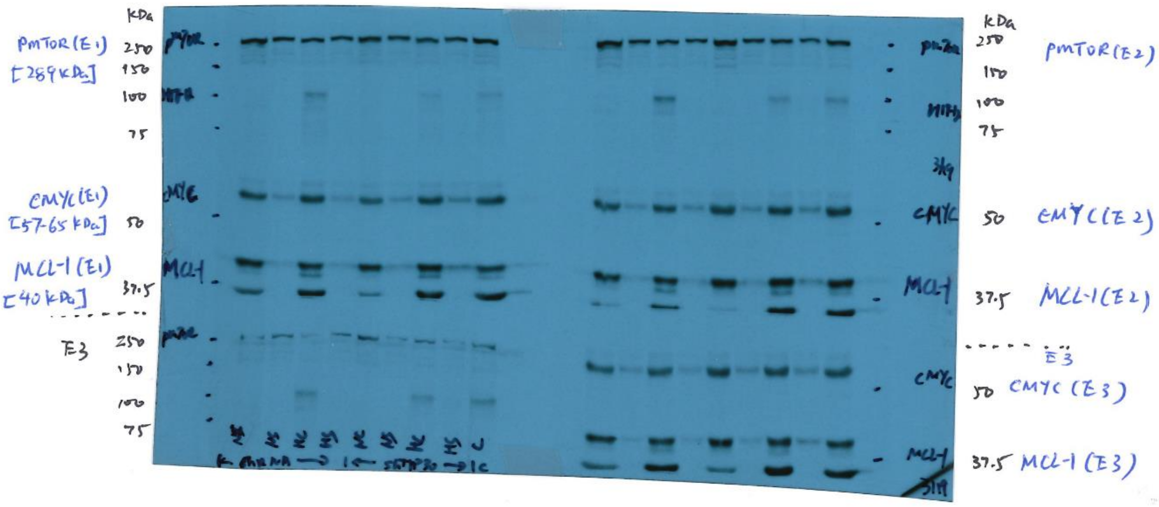

Supplemental Figure S3-3-3. Uncropped Western blot images showing the expression of phospho-mTOR (S2448; 289 kDa), c-Myc (57~65 kDa) and MCL-1 (40 kDa) in A549shNT and A549shHTATIP2 cell lysates.

***IN VITRO* HTATIP2 knockdown study**

A549shNT versus A549shHTATIP2 cell line under hypoxia (0.5% O2) and normoxia condition in the presence and absence of 10 μM sorafenib for 24 hours in DMEM containing 0.5% FBS with high glucose and L-glutamine and without sodium pyruvate. Samples were from three independent experiments [E1, E2 and E3]
From Left to Right:
(#1 ~4) A549shNT: Normoxia_control (NC), Normoxia_sorafenib (NS), Hypoxia_control (HC), Hypoxia_sorafenib (HS); (#5 ~8) A549shNT: Normoxia_control (NC), Normoxia_sorafenib (NS), Hypoxia_control (HC), Hypoxia_sorafenib (HS); #9: positive control.

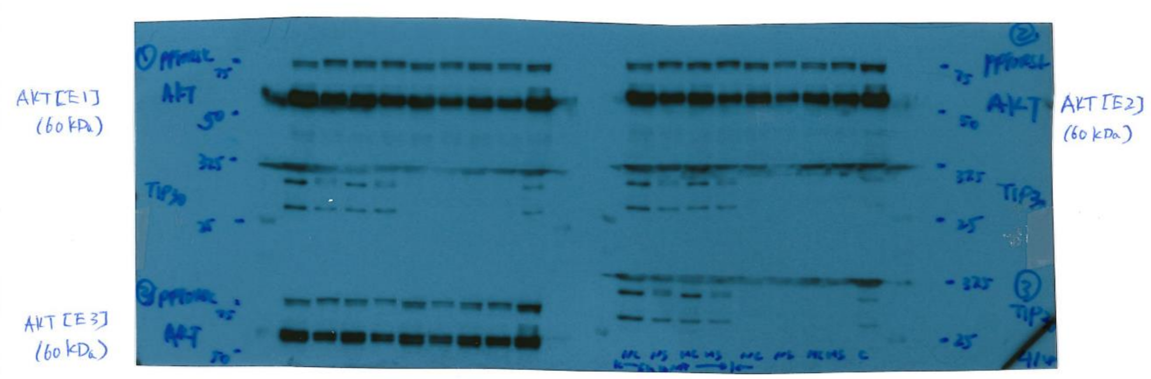

Supplemental Figure S3-3-4. Uncropped Western blot images showing the expression of Akt (60 kDa) in A549shNT and A549shHTATIP2 cell lysates.

IN VITRO **HTATIP2 knockdown study**

A549shNT versus A549shHTATIP2 cell line under hypoxia (0.5% O2) and normoxia condition in the presence and absence of 10 µM sorafenib for 24 hours in DMEM containing 0.5% FBS with high glucose and L-glutamine and without sodium pyruvate. Samples were from three independent experiments [E1, E2 and E3]
From Left to Right:
(#1 ~4) A549shNT: Normoxia_control (NC), Normoxia_sorafenib (NS), Hypoxia_control (HC), Hypoxia_sorafenib (HS); (#5 ~8) A549shNT: Normoxia_control (NC), Normoxia_sorafenib (NS), Hypoxia_control (HC), Hypoxia_sorafenib (HS); #9: positive control.

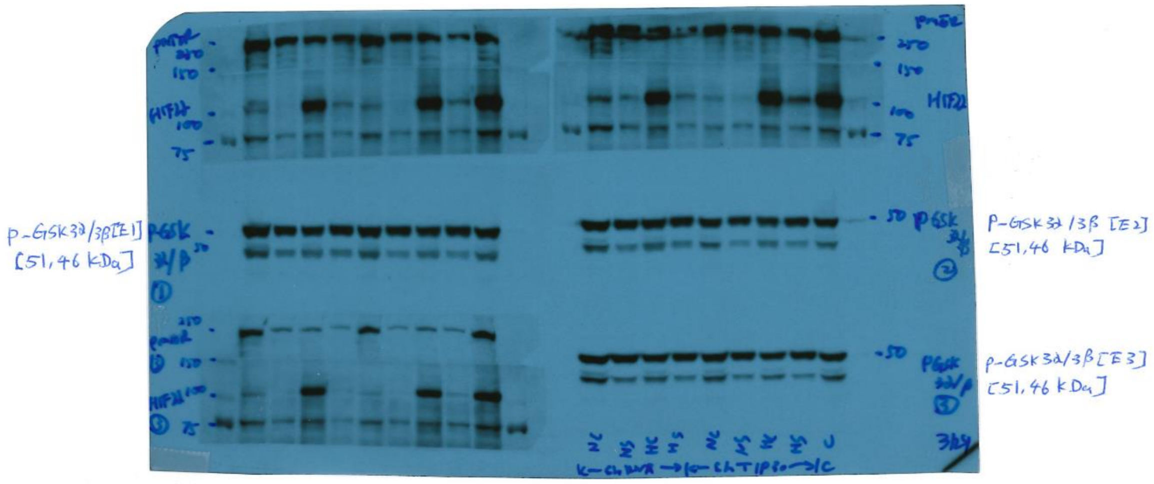

Supplemental Figure S3-3-5. Uncropped Western blot images showing the expression of phospho-GSK3α/3β (S21/S9; 51, 46 kDa) in A549shNT and A549shHTATIP2 cell lysates.

***IN VITRO* HTATIP2 knockdown study**

A549shNT versus A549shHTATIP2 cell line under hypoxia (0.5% O2) and normoxia condition in the presence and absence of 10 μM sorafenib for 24 hours in DMEM containing 0.5% FBS with high glucose and L-glutamine and without sodium pyruvate. Samples were from three independent experiments [E1, E2 and E3]
From Left to Right:
(#1 ~4) A549shNT: Normoxia_control (NC), Normoxia_sorafenib (NS), Hypoxia_control (HC), Hypoxia_sorafenib (HS); (#5 ~8) A549shNT: Normoxia_control (NC), Normoxia_sorafenib (NS), Hypoxia_control (HC), Hypoxia_sorafenib (HS); #9: positive control.

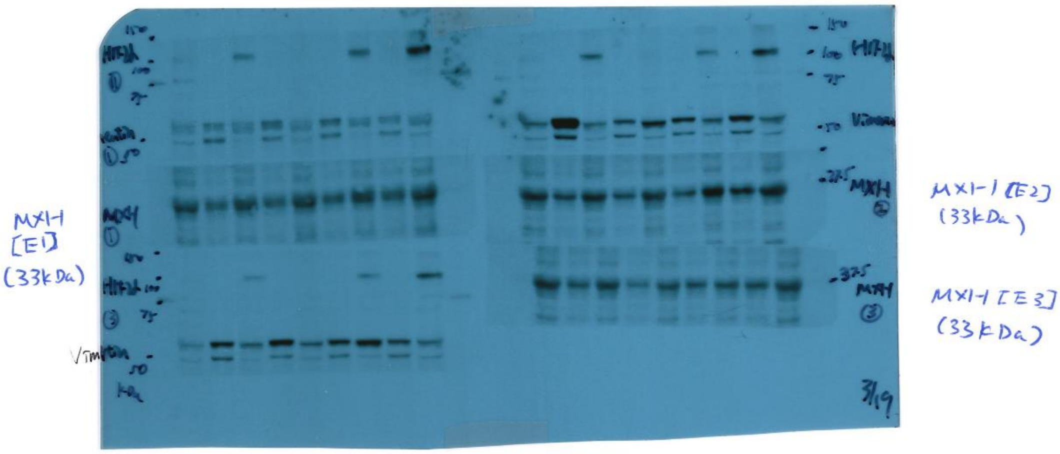

Supplemental Figure S3-3-6. Uncropped Western blot images showing the expression of MXI1 (33 kDa) in A549shNT and A549shHTATIP2 cell lysates.

IN VITRO **HTATIP2 knockdown study**

A549shNT versus A549shHTATIP2 cell line under hypoxia (0.5% O2) and normoxia condition in the presence and absence of 10 μM sorafenib for 24 hours in DMEM containing 0.5% FBS with high glucose and L-glutamine and without sodium pyruvate. Samples were from three independent experiments [E1, E2 and E3]
From Left to Right:
(#1 ~4) A549shNT: Normoxia_control (NC), Normoxia_sorafenib (NS), Hypoxia_control (HC), Hypoxia_sorafenib (HS); (#5 ~8) A549shNT: Normoxia_control (NC), Normoxia_sorafenib (NS), Hypoxia_control (HC), Hypoxia_sorafenib (HS); #9: positive control.

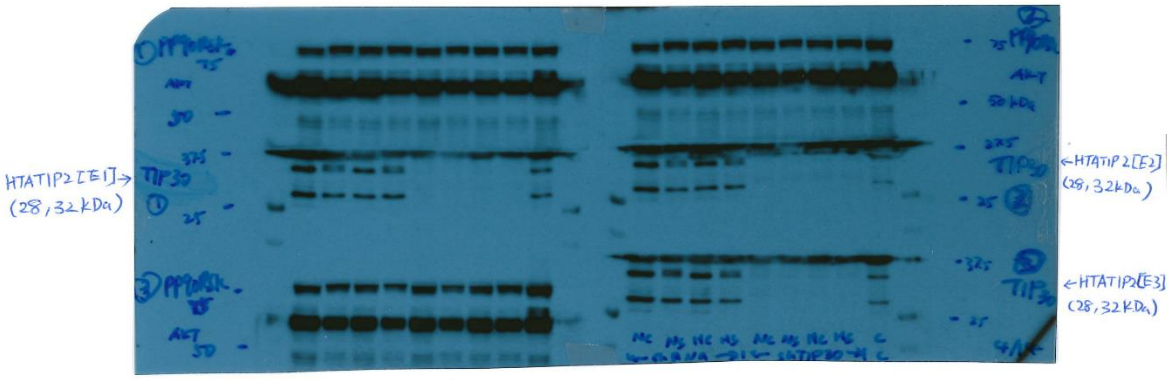

Supplemental Figure S3-3-7. Uncropped Western blot images showing the expression of HTATIP2 (28, 32 kDa) in A549shNT and A549shHTATIP2 cell lysates.

IN VITRO **HTATIP2 knockdown study**

A549shNT versus A549shHTATIP2 cell line under hypoxia (0.5% O2) and normoxia condition in the presence and absence of 10 μM sorafenib for 24 hours in DMEM containing 0.5% FBS with high glucose and L-glutamine and without sodium pyruvate. Samples were from three independent experiments [E1, E2 and E3]
From Left to Right:
(#1 ~4) A549shNT: Normoxia_control (NC), Normoxia_sorafenib (NS), Hypoxia_control (HC), Hypoxia_sorafenib (HS); (#5 ~8) A549shNT: Normoxia_control (NC), Normoxia_sorafenib (NS), Hypoxia_control (HC), Hypoxia_sorafenib (HS); #9: positive control.

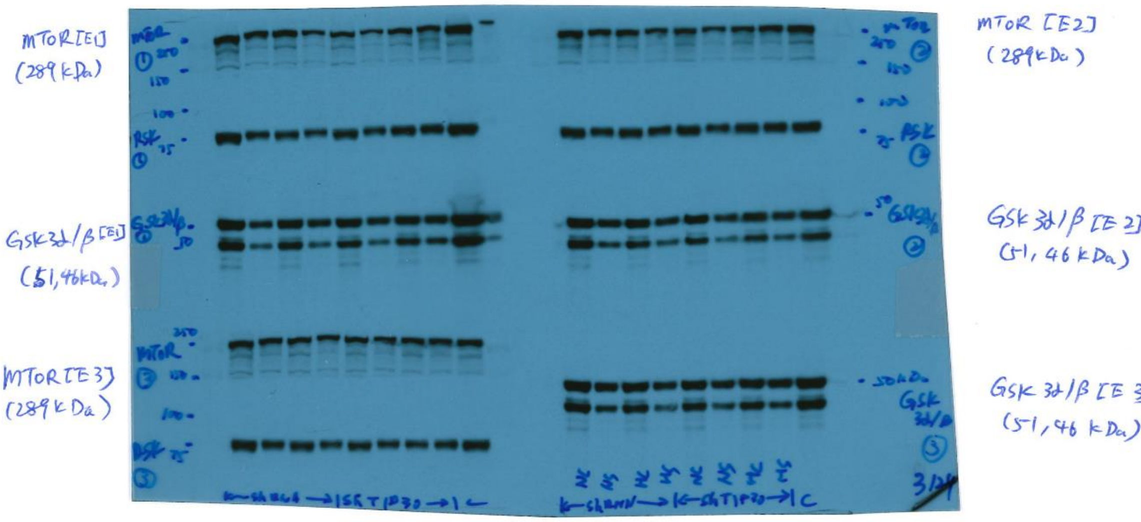

Supplemental Figure S3-3-8. Uncropped Western blot images showing the expression of mTOR (289 kDa) and GSK3α/3β (51, 46 kDa) in A549shNT and A549shHTATIP2 cell lysates.

IN VITRO **HTATIP2 knockdown study**

A549shNT versus A549shHTATIP2 cell line under hypoxia (0.5% O2) and normoxia condition in the presence and absence of 10 μM sorafenib for 24 hours in DMEM containing 0.5% FBS with high glucose and L-glutamine and without sodium pyruvate. Samples were from three independent experiments [E1, E2 and E3]
From Left to Right:
(#1 ~4) A549shNT: Normoxia_control (NC), Normoxia_sorafenib (NS), Hypoxia_control (HC), Hypoxia_sorafenib (HS); (#5 ~8) A549shNT: Normoxia_control (NC), Normoxia_sorafenib (NS), Hypoxia_control (HC), Hypoxia_sorafenib (HS); #9: positive control.

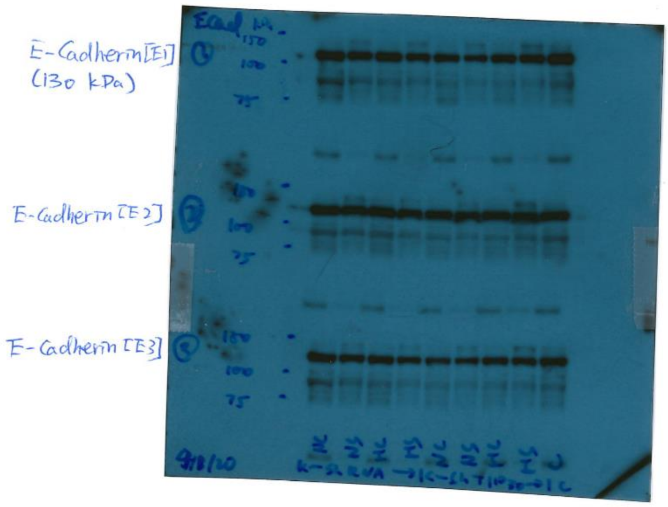

Supplemental Figure S3-3-9. Uncropped Western blot images showing the expression of E-Cadherin (130 kDa) in A549shNT and A549shHTATIP2 cell lysates.

IN VITRO **HTATIP2 knockdown study**

A549shNT versus A549shHTATIP2 cell line under hypoxia (0.5% O2) and normoxia condition in the presence and absence of 10 μM sorafenib for 24 hours in DMEM containing 0.5% FBS with high glucose and L-glutamine and without sodium pyruvate. Samples were from three independent experiments [E1, E2 and E3]
From Left to Right:
(#1 ~4) A549shNT: Normoxia_control (NC), Normoxia_sorafenib (NS), Hypoxia_control (HC), Hypoxia_sorafenib (HS); (#5 ~8) A549shNT: Normoxia_control (NC), Normoxia_sorafenib (NS), Hypoxia_control (HC), Hypoxia_sorafenib (HS); #9: positive control.

β-catenin [E1] (92kDa)

β-catenin (E2) (92kDa)

β-catenin (E3) (92kDa)

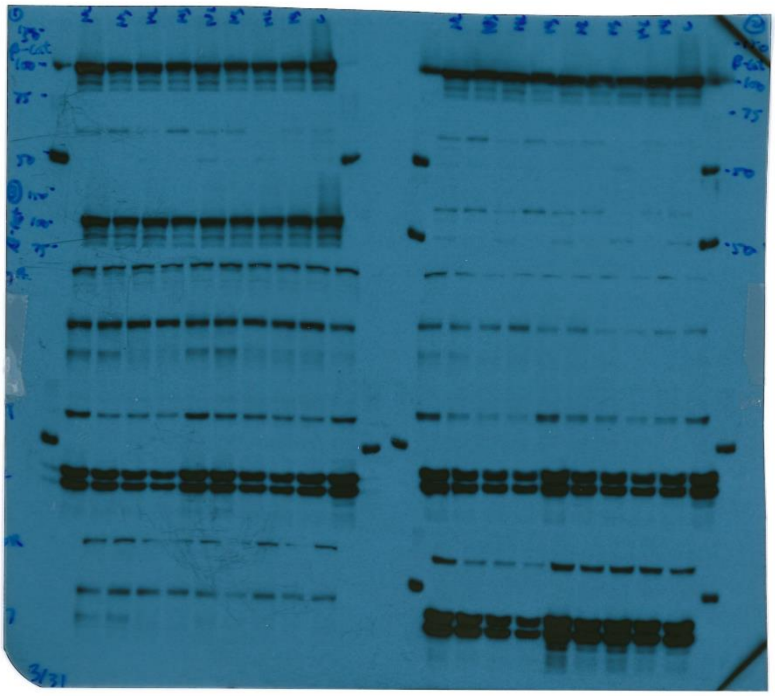

Supplemental Figure S3-3-10. Uncropped Western blot images showing the expression of β–Catenin (92 kDa) in A549shNT and A549shHTATIP2 cell lysates.

IN VITRO **HTATIP2 knockdown study**

A549shNT versus A549shHTATIP2 cell line under hypoxia (0.5% O2) and normoxia condition in the presence and absence of 10 μM sorafenib for 24 hours in DMEM containing 0.5% FBS with high glucose and L-glutamine and without sodium pyruvate. Samples were from three independent experiments [E1, E2 and E3]
From Left to Right:
(#1 ~4) A549shNT: Normoxia_control (NC), Normoxia_sorafenib (NS), Hypoxia_control (HC), Hypoxia_sorafenib (HS); (#5 ~8) A549shNT: Normoxia_control (NC), Normoxia_sorafenib (NS), Hypoxia_control (HC), Hypoxia_sorafenib (HS); #9: positive control.

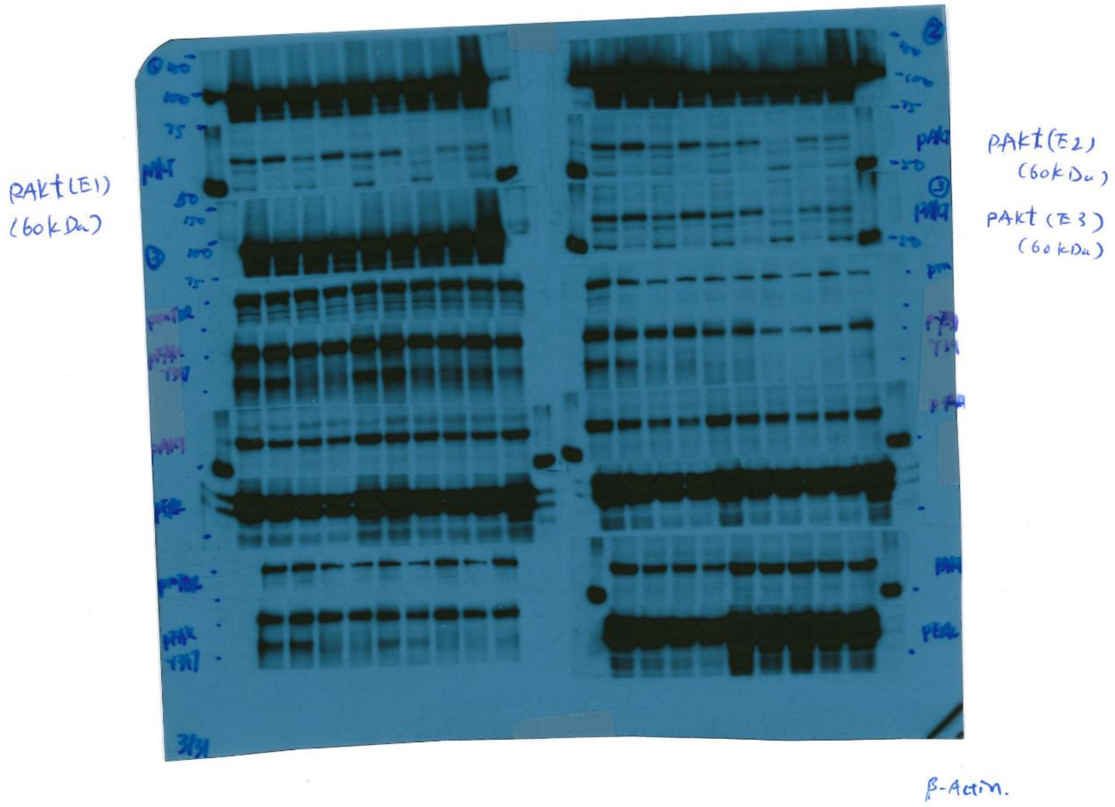

Supplemental Figure S3-3-11. Uncropped Western blot images showing the expression of phospho-Akt (S473; 60 kDa) in A549shNT and A549shHTATIP2 cell lysates.

***IN VITRO* HTATIP2 knockdown study**

A549shNT versus A549shHTATIP2 cell line under hypoxia (0.5% O2) and normoxia condition in the presence and absence of 10 μM sorafenib for 24 hours in DMEM containing 0.5% FBS with high glucose and L-glutamine and without sodium pyruvate. Samples were from three independent experiments [E1, E2 and E3]
From Left to Right:
(#1 ~4) A549shNT: Normoxia_control (NC), Normoxia_sorafenib (NS), Hypoxia_control (HC), Hypoxia_sorafenib (HS); (#5 ~8) A549shNT: Normoxia_control (NC), Normoxia_sorafenib (NS), Hypoxia_control (HC), Hypoxia_sorafenib (HS); #9: positive control.

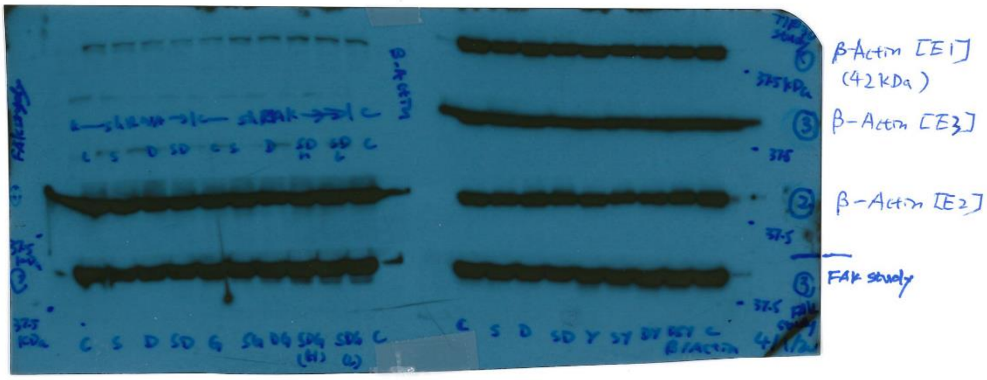

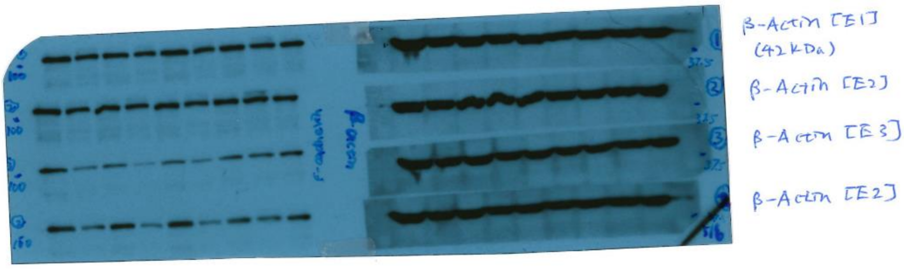

Supplemental Figure S3-3-12. Uncropped Western blot images showing the expression of β–Actin (42 kDa) in A549shNT and A549shHTATIP2 cell lysates.

HTATIP2 knockdown *IN VIVO* study
Mouse ID: Sorafenib group: #1-1, #2-1, #3-1, #3-2, #5-1, #5-2.
Control group: #4-1, #4-2, #4-3, #4-4, #6-1, #6-2.
A – A549 parental tumors, T – A549shHTATIP2 tumors
From Left to Right:
Membrane 1: A1-1, T1-1, A2-1, T2-1, A3-1, T3-1, A4-1, T4-1, A4-2, T4-2, A4-3, T4-3, positive control
Membrane 2: A3-2, T3-2, A5-1, T5-1, A5-2, T5-2, A4-4, T4-4, A6-1, T6-1, A6-2, T6-2, positive control

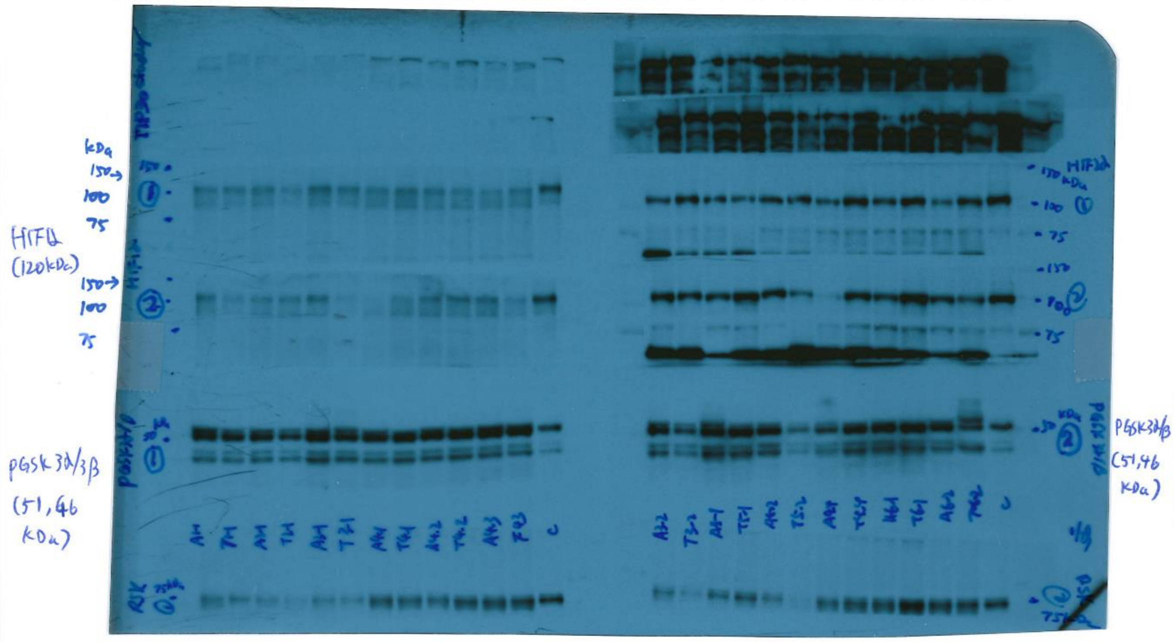

Supplemental Figure S3-4-1. Uncropped Western blot images showing the expression of HIF1α (120 kDa) and phospho-GSK3α/3β (S21/S9; 51, 46 kDa) in A549 parental and A549shHTATIP2 tumor homogenates.

HTATIP2 knockdown *IN VIVO* study

Mouse ID: Sorafenib group: #1-1, #2-1, #3-1, #3-2, #5-1, #5-2.
Control group: #4-1, #4-2, #4-3, #4-4, #6-1, #6-2.

A – A549 parental tumors, T – A549shHTATIP2 tumors

From Left to Right:

Membrane 1: A1-1, T1-1, A2-1, T2-1, A3-1, T3-1, A4-1, T4-1, A4-2, T4-2, A4-3, T4-3, positive control

Membrane 2: A3-2, T3-2, A5-1, T5-1, A5-2, T5-2, A4-4, T4-4, A6-1, T6-1, A6-2, T6-2, positive control

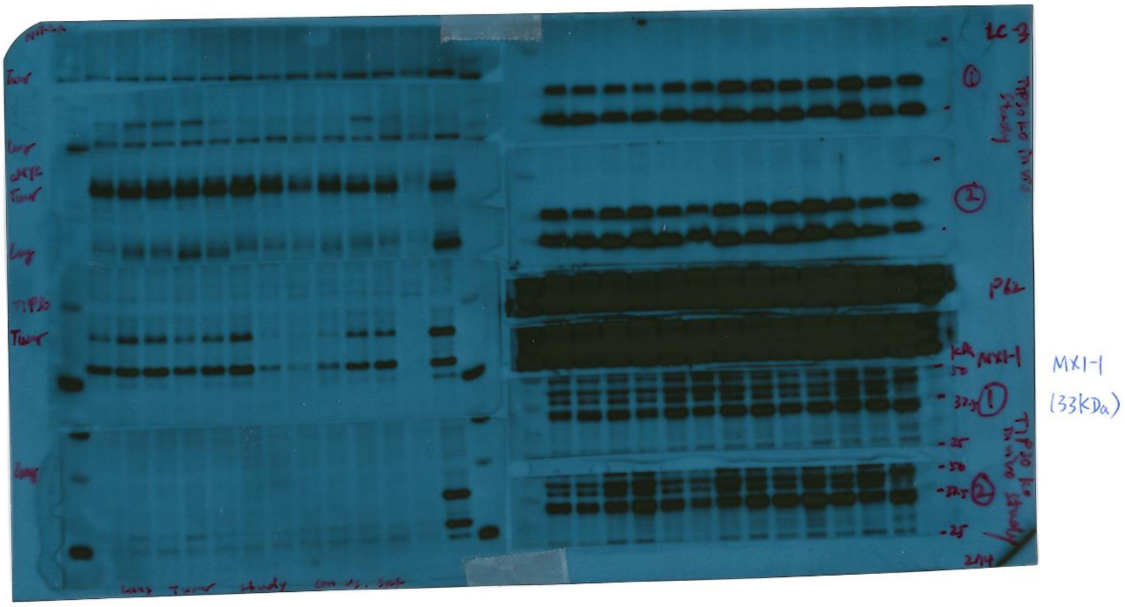

Supplemental Figure S3-4-2. Uncropped Western blot images showing the expression of MXI1 (33 kDa) in A549 parental and A549shHTATIP2 tumor homogenates.

HTATIP2 knockdown *IN VIVO* study
Mouse ID: Sorafenib group: #1-1, #2-1, #3-1, #3-2, #5-1, #5-2.
Control group: #4-1, #4-2, #4-3, #4-4, #6-1, #6-2.
A – A549 parental tumors, T – A549shHTATIP2 tumors
From Left to Right:
Membrane 1: A1-1, T1-1, A2-1, T2-1, A3-1, T3-1, A4-1, T4-1, A4-2, T4-2, A4-3, T4-3, positive control
Membrane 2: A3-2, T3-2, A5-1, T5-1, A5-2, T5-2, A4-4, T4-4, A6-1, T6-1, A6-2, T6-2, positive control

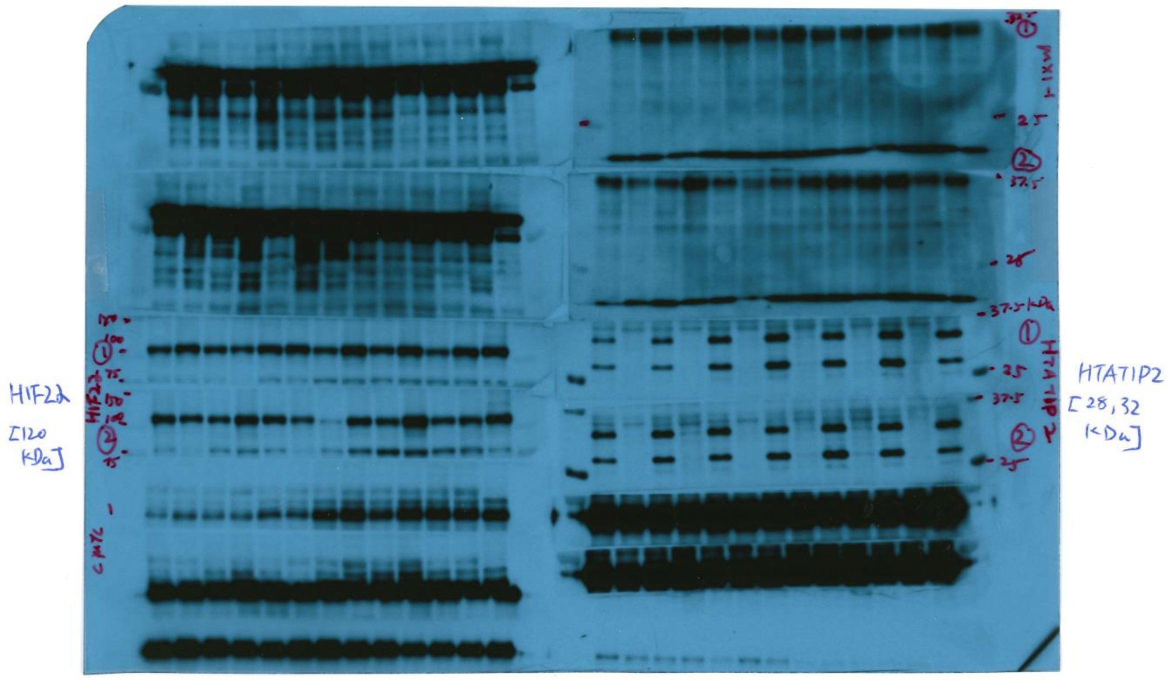

Supplemental Figure S3-4-3. Uncropped Western blot images showing the expression of HIF2α (120 kDa) and HTATIP2 (28, 32 kDa) in A549 parental and A549shHTATIP2 tumor homogenates.

HTATIP2 knockdown *IN VIVO* study
Mouse ID: Sorafenib group: #1-1, #2-1, #3-1, #3-2, #5-1, #5-2.
Control group: #4-1, #4-2, #4-3, #4-4, #6-1, #6-2.
A – A549 parental tumors, T – A549shHTATIP2 tumors
From Left to Right:
Membrane 1: A1-1, T1-1, A2-1, T2-1, A3-1, T3-1, A4-1, T4-1, A4-2, T4-2, A4-3, T4-3, positive control
Membrane 2: A3-2, T3-2, A5-1, T5-1, A5-2, T5-2, A4-4, T4-4, A6-1, T6-1, A6-2, T6-2, positive control

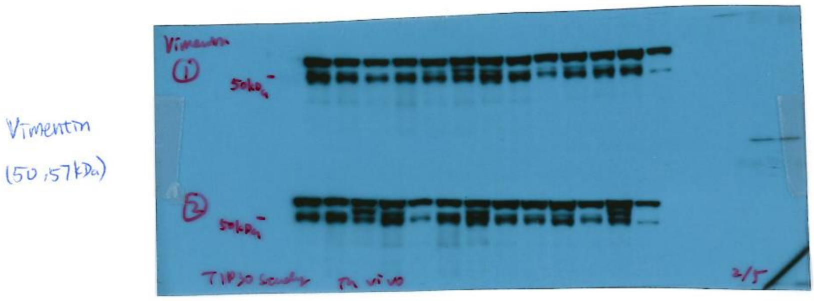

Supplemental Figure S3-4-4. Uncropped Western blot images showing the expression of Vimentin (50, 57 kDa) in A549 parental and A549shHTATIP2 tumor homogenates.

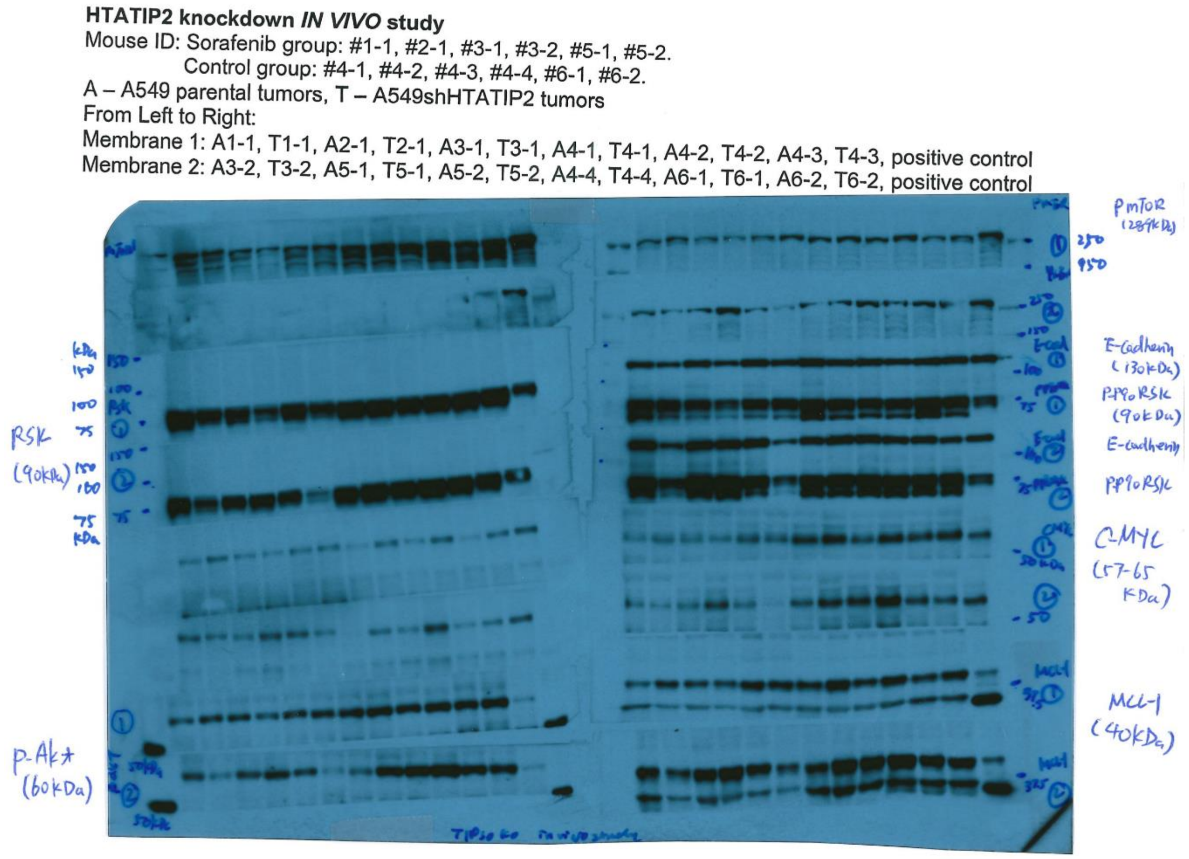

Supplemental Figure S3-4-5. Uncropped Western blot images showing the expression of phospho-mTOR (S2448; 289 kDa), E-Cadherin (130 kDa), RSK (90 kDa), phospho-p90RSK (S380; 90 kDa), phospho-Akt (S473; 60 kDa), c-Myc (57 ~ 65 kDa), MCL-1 (40 kDa) in A549 parental and A549shHTATIP2 tumor homogenates.

HTATIP2 knockdown *IN VIVO* study

Mouse ID: Sorafenib group: #1-1, #2-1, #3-1, #3-2, #5-1, #5-2.

Control group: #4-1, #4-2, #4-3, #4-4, #6-1, #6-2.

A – A549 parental tumors, T – A549shHTATIP2 tumors

From Left to Right:

Membrane 1: A1-1, T1-1, A2-1, T2-1, A3-1, T3-1, A4-1, T4-1, A4-2, T4-2, A4-3, T4-3, positive control

Membrane 2: A3-2, T3-2, A5-1, T5-1, A5-2, T5-2, A4-4, T4-4, A6-1, T6-1, A6-2, T6-2, positive control

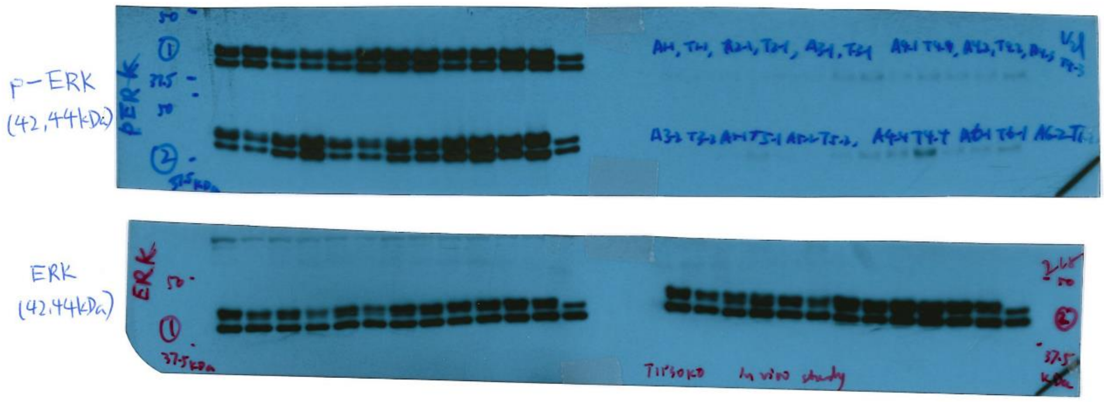

Supplemental Figure S3-4-6. Uncropped Western blot images showing the expression of ERK1/2 (42, 44 kDa) and phospho-ERK1/2 (T202/Y204; 42, 44 kDa) in A549 parental and A549shHTATIP2 tumor homogenates.

HTATIP2 knockdown *IN VIVO* study

Mouse ID: Sorafenib group: #1-1, #2-1, #3-1, #3-2, #5-1, #5-2.

Control group: #4-1, #4-2, #4-3, #4-4, #6-1, #6-2.

A – A549 parental tumors, T – A549shHTATIP2 tumors

From Left to Right:

Membrane 1: A1-1, T1-1, A2-1, T2-1, A3-1, T3-1, A4-1, T4-1, A4-2, T4-2, A4-3, T4-3, positive control

Membrane 2: A3-2, T3-2, A5-1, T5-1, A5-2, T5-2, A4-4, T4-4, A6-1, T6-1, A6-2, T6-2, positive control

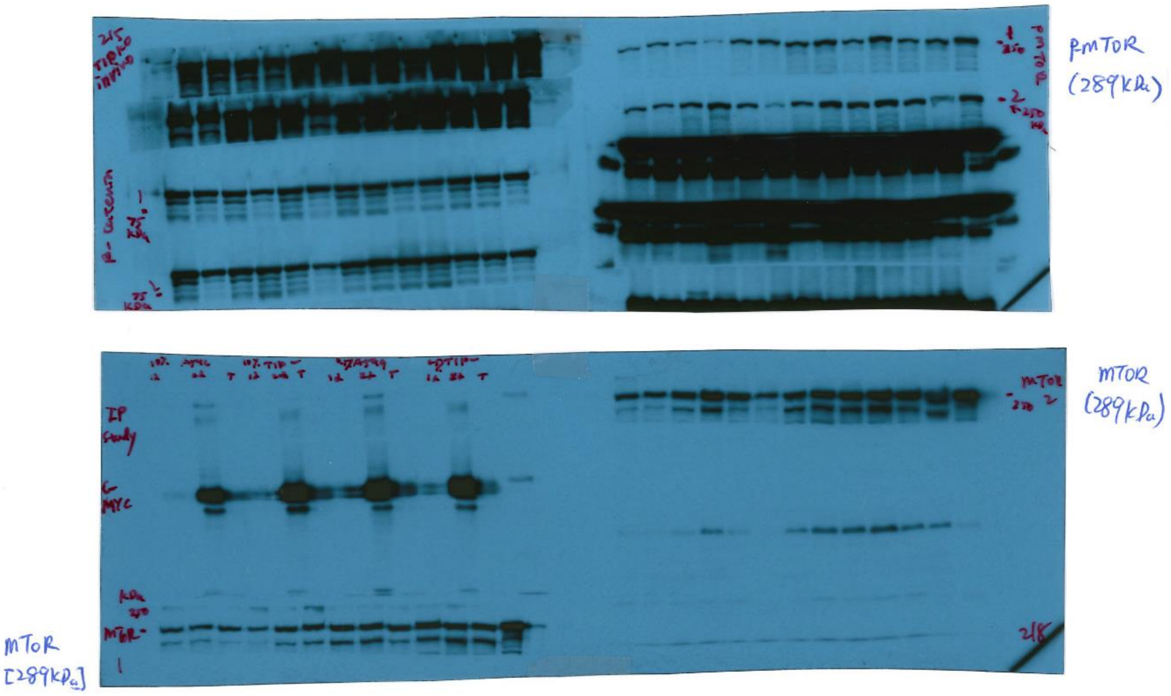

Supplemental Figure S3-4-7. Uncropped Western blot images showing the expression of mTOR (289 kDa) and phospho-mTOR (S2448; 289 kDa) in A549 parental and A549shHTATIP2 tumor homogenates.

HTATIP2 knockdown *IN VIVO* study
Mouse ID: Sorafenib group: #1-1, #2-1, #3-1, #3-2, #5-1, #5-2.
Control group: #4-1, #4-2, #4-3, #4-4, #6-1, #6-2.
A – A549 parental tumors, T – A549shHTATIP2 tumors
From Left to Right:
Membrane 1: A1-1, T1-1, A2-1, T2-1, A3-1, T3-1, A4-1, T4-1, A4-2, T4-2, A4-3, T4-3, positive control
Membrane 2: A3-2, T3-2, A5-1, T5-1, A5-2, T5-2, A4-4, T4-4, A6-1, T6-1, A6-2, T6-2, positive control

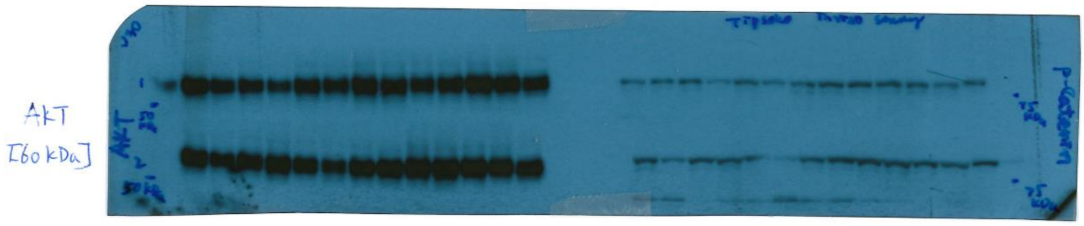

Supplemental Figure S3-4-8. Uncropped Western blot images showing the expression of Akt (60 kDa) in A549 parental and A549shHTATIP2 tumor homogenates.

HTATIP2 knockdown *IN VIVO* study
Mouse ID: Sorafenib group: #1-1, #2-1, #3-1, #3-2, #5-1, #5-2.
Control group: #4-1, #4-2, #4-3, #4-4, #6-1, #6-2.
A – A549 parental tumors, T – A549shHTATIP2 tumors
From Left to Right:
Membrane 1: A1-1, T1-1, A2-1, T2-1, A3-1, T3-1, A4-1, T4-1, A4-2, T4-2, A4-3, T4-3, positive control
Membrane 2: A3-2, T3-2, A5-1, T5-1, A5-2, T5-2, A4-4, T4-4, A6-1, T6-1, A6-2, T6-2, positive control

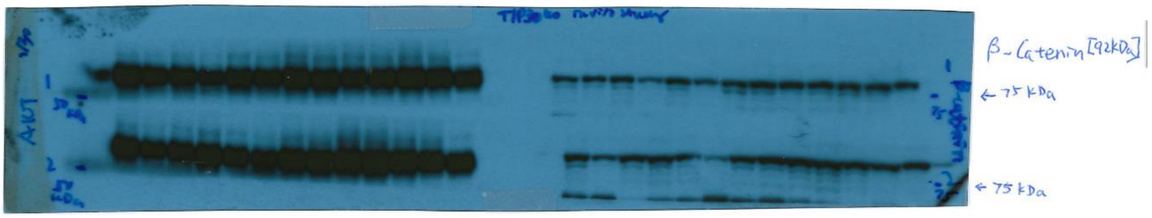

Supplemental Figure S3-4-9. Uncropped Western blot images showing the expression of β-Catenin (92 kDa) in A549 parental and A549shHTATIP2 tumor homogenates.

HTATIP2 knockdown *IN VIVO* study

Mouse ID: Sorafenib group: #1-1, #2-1, #3-1, #3-2, #5-1, #5-2.
Control group: #4-1, #4-2, #4-3, #4-4, #6-1, #6-2.

A – A549 parental tumors, T – A549shHTATIP2 tumors

From Left to Right:

Membrane 1: A1-1, T1-1, A2-1, T2-1, A3-1, T3-1, A4-1, T4-1, A4-2, T4-2, A4-3, T4-3, positive control

Membrane 2: A3-2, T3-2, A5-1, T5-1, A5-2, T5-2, A4-4, T4-4, A6-1, T6-1, A6-2, T6-2, positive control

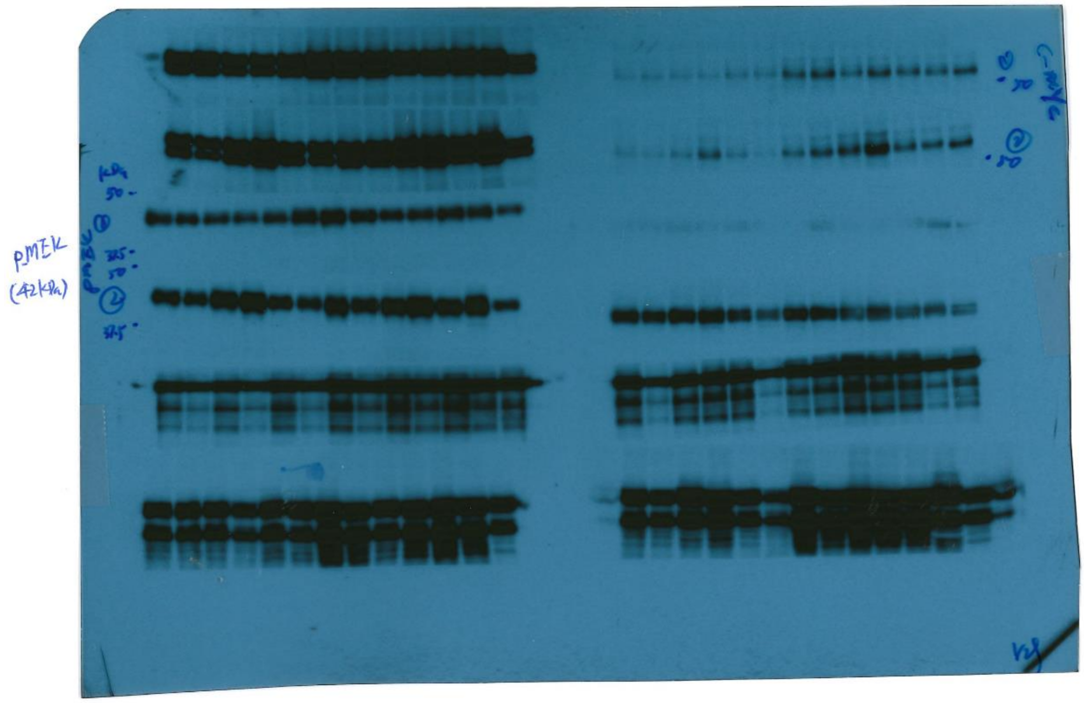

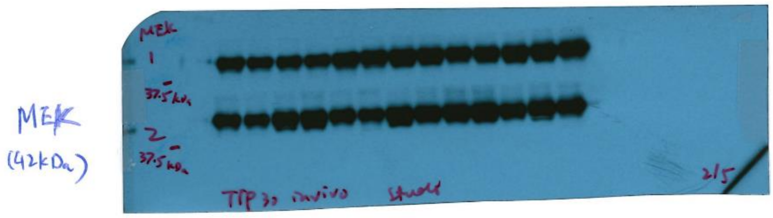

Supplemental Figure S3-4-10. Uncropped Western blot images showing the expression of MEK (42 kDa) and phospho-MEK (S217/221; 42 kDa) in A549 parental and A549shHTATIP2 tumor homogenates.

HTATIP2 knockdown *IN VIVO* study
Mouse ID: Sorafenib group: #1-1, #2-1, #3-1, #3-2, #5-1, #5-2.
Control group: #4-1, #4-2, #4-3, #4-4, #6-1, #6-2.
A – A549 parental tumors, T – A549shHTATIP2 tumors
From Left to Right:
Membrane 1: A1-1, T1-1, A2-1, T2-1, A3-1, T3-1, A4-1, T4-1, A4-2, T4-2, A4-3, T4-3, positive control
Membrane 2: A3-2, T3-2, A5-1, T5-1, A5-2, T5-2, A4-4, T4-4, A6-1, T6-1, A6-2, T6-2, positive control

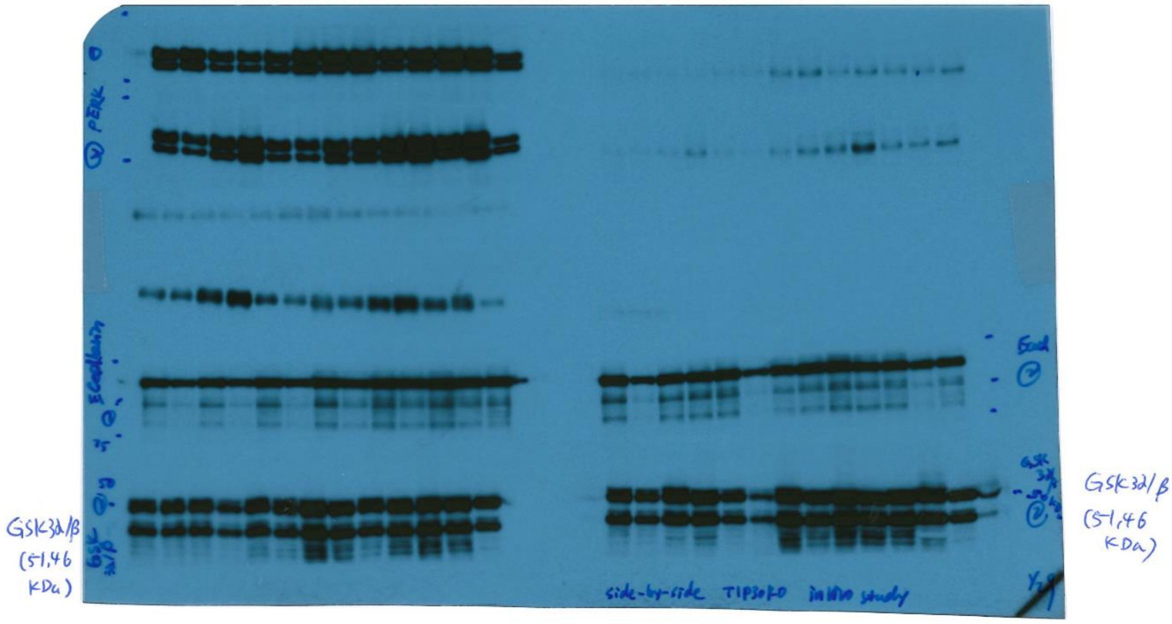

Supplemental Figure S3-4-11. Uncropped Western blot images showing the expression of GSK3α/3β (51, 46 kDa) in A549 parental and A549shHTATIP2 tumor homogenates.

HTATIP2 knockdown *IN VIVO* study
Mouse ID: Sorafenib group: #1-1, #2-1, #3-1, #3-2, #5-1, #5-2.
Control group: #4-1, #4-2, #4-3, #4-4, #6-1, #6-2.
A – A549 parental tumors, T – A549shHTATIP2 tumors
From Left to Right:
Membrane 1: A1-1, T1-1, A2-1, T2-1, A3-1, T3-1, A4-1, T4-1, A4-2, T4-2, A4-3, T4-3, positive control
Membrane 2: A3-2, T3-2, A5-1, T5-1, A5-2, T5-2, A4-4, T4-4, A6-1, T6-1, A6-2, T6-2, positive control

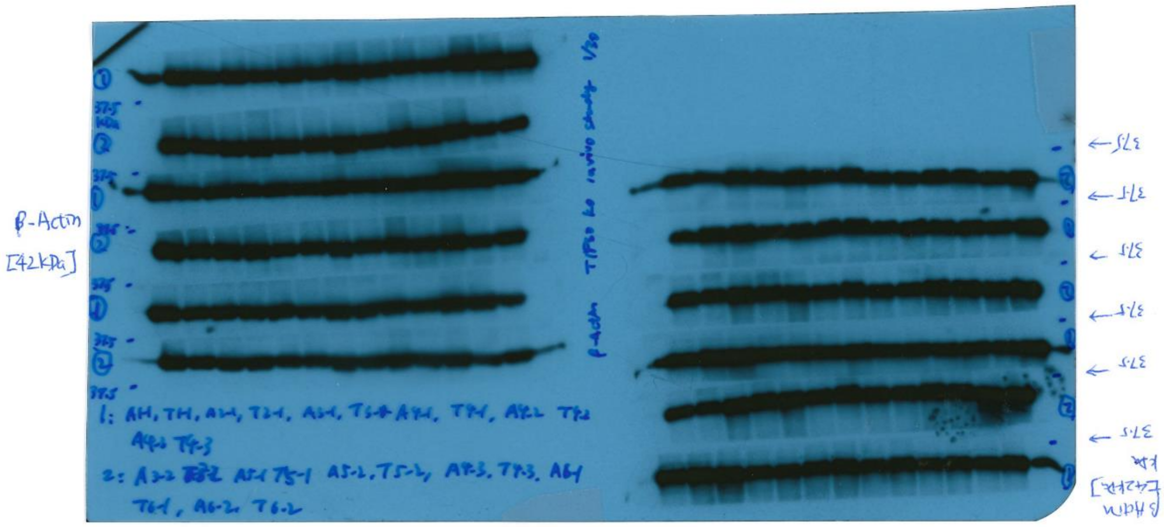

Supplemental Figure S3-4-12. Uncropped Western blot images showing the expression of β-Actin (42 kDa) in A549 parental and A549shHTATIP2 tumor homogenates.
